# Individualizing Perioperative Analgesia: Patient-Specific Determinants of Postoperative Pain and Opioid-Related Outcomes

**DOI:** 10.3390/medicina62091789

**Published:** 2026-09-17

**Authors:** Sarah Kazemeini, Ryan Shih, Sunny Zhang, Raphael Cohen, Nathaniel LaBarre, Moonis Ghani, George Tsao

**Affiliations:** 1 Kirk Kerkorian School of Medicine at UNLV, University of Nevada Las Vegas, 625 Shadow Lane, Las Vegas, NV 89106, USA; 2 North Las Vegas VA Medical Center, 6900 N. Pecos Road, Las Vegas, NV 89086, USA

**Keywords:** postoperative pain, perioperative analgesia, opioids, biological sex, frailty, chronic pain, opioid tolerance

## Abstract

*Background and Objectives*: Postoperative pain and opioid requirements can vary widely, even among patients undergoing similar procedures. This narrative review examines how biological sex, age, frailty, chronic pain, preoperative opioid exposure, and psychological factors may affect postoperative pain, opioid response, and recovery. *Materials and Methods*: PubMed and Google Scholar were searched from database inception through 18 June 2026. Relevant systematic reviews, meta-analyses, clinical guidelines, randomized trials, and observational studies involving adult surgical patients were included, with a focus on postoperative pain, opioid use, adverse effects, persistent pain, and functional recovery. Human perioperative evidence was prioritized, while experimental and preclinical evidence was used selectively to explain relevant biological mechanisms. *Results*: The available evidence was heterogeneous across patient populations, surgical procedures, analgesic techniques, and outcome definitions. No single patient characteristic consistently predicts postoperative pain or opioid requirements. Female sex is associated with a greater risk of postoperative nausea and vomiting, but reported differences in pain and opioid efficacy are inconsistent. Older and frail patients may be more susceptible to sedation, respiratory depression, delirium, and other opioid-related adverse effects. Patients with chronic pain or prior opioid exposure often have more difficult postoperative pain control because of tolerance, dependence, or possible opioid-induced hyperalgesia. Anxiety, depression, and pain catastrophizing may also contribute to greater pain and poorer recovery, although their relationship with opioid consumption is less consistent. *Conclusions*: Perioperative analgesia should be based on the patient’s overall clinical picture rather than any single risk factor. The proposed framework is conceptual and has not been clinically validated. Considering these characteristics together may help clinicians select appropriate multimodal treatments, anticipate analgesic needs, monitor for adverse effects, and support recovery.

## 1. Introduction

Postoperative pain remains a major clinical challenge and one of the most common sources of morbidity after surgery [1]. Although acute postoperative pain is expected after tissue injury, inadequate control can prolong recovery, worsen patient satisfaction, and contribute to later chronic pain [2]. Even with modern perioperative care, many surgical patients still report moderate to severe pain, and large numbers of operations worldwide mean that the burden extends across health systems rather than isolated procedures [3]. Chronic postsurgical pain (CPSP) is especially important because it can persist well beyond the normal healing period and remain clinically meaningful months after surgery [4,5,6]. Chronic postsurgical pain (CPSP) is defined as pain that develops or increases after surgery, persists beyond the expected healing period for at least three months, and is not better explained by another condition [5].

Over the past several decades, perioperative pain management has shifted from opioid-centered analgesia toward multimodal strategies designed to improve recovery [7]. Current reviews describe multimodal analgesia as the use of two or more systemic analgesics, often combined with regional anesthesia when possible [8,9]. The fact that multimodal protocols are now standard, however, does not mean they fully solve the problem. Pain trajectories still vary significantly between patients, procedures, and settings [10,11].

Despite standardized perioperative pathways, postoperative pain and opioid requirements remain highly variable between patients undergoing similar surgical procedures [12]. This variability reflects the complex interaction of patient-specific biological, psychological, and clinical factors rather than surgical characteristics alone [13,14]. Numerous studies have identified characteristics such as age, biological sex, pre-existing chronic pain, opioid tolerance, and psychiatric comorbidities as important contributors to postoperative analgesic response [15,16,17,18]. While many of these factors are recognized individually, they are often considered in isolation despite their collective influence on perioperative pain management and opioid-related outcomes.

Postoperative pain and opioid responsiveness emerge from the interaction of multiple patient-specific biological, clinical, and psychological characteristics that collectively influence analgesic efficacy, adverse effects, and recovery [19,20]. This narrative review synthesizes these interacting determinants into a clinically applicable framework for individualized perioperative pain management, emphasizing integrated risk assessment to guide analgesic selection, opioid stewardship, postoperative monitoring, and functional recovery.

## 2. Scope and Methodology

This narrative review was designed to examine how commonly encountered patient characteristics influence postoperative pain, opioid-related outcomes, and functional recovery. Biological sex, age, frailty, chronic pain, preoperative opioid exposure, and psychological factors were selected because they are frequently identified during preoperative assessment and may affect both analgesic requirements and the risk of treatment-related adverse effects.

PubMed and Google Scholar were searched from database inception through 18 June 2026. Search terms included “postoperative pain,” “perioperative analgesia,” “opioid response,” “biological sex,” “sex differences,” “age,” “frailty,” “chronic pain,” “preoperative opioid use,” “opioid tolerance,” “opioid-induced hyperalgesia,” “anxiety,” “depression,” and “pain catastrophizing.” A representative PubMed search combined perioperative terms with the patient-specific factors as follows: (“postoperative pain” OR “perioperative analgesia” OR “postoperative opioid use” OR “opioid response”) AND (“biological sex” OR “sex differences” OR age OR frailty OR “chronic pain” OR “preoperative opioid use” OR “opioid tolerance” OR “opioid-induced hyperalgesia” OR anxiety OR depression OR “pain catastrophizing”). Google Scholar was searched using similar combinations of terms, with results reviewed in order of relevance.

Two authors (S.K. and R.S.) screened titles and abstracts and reviewed the full texts of potentially relevant articles. Differences regarding inclusion were resolved through discussion. Duplicate articles were identified manually using the title, authors, publication year, and DOI. Because this was a narrative review, the number of initial search results and Google Scholar pages screened was not prospectively recorded. Approximately 140 publications were included in the final synthesis.

Articles were considered relevant if they addressed postoperative pain intensity, analgesic or opioid consumption, opioid-related adverse effects, chronic postsurgical pain, persistent postoperative opioid use, or functional recovery in adult surgical patients. Systematic reviews, meta-analyses, clinical guidelines, randomized trials, and observational studies were prioritized. Human perioperative evidence was used whenever available, while experimental and preclinical studies were included selectively to explain relevant biological mechanisms. Non-English publications, non-peer-reviewed sources, and articles without clear relevance to perioperative pain management were excluded. Reference lists were reviewed selectively to identify additional relevant studies.

Evidence was assessed qualitatively rather than through a formal grading system. Systematic reviews, meta-analyses, and clinical guidelines were given greater weight when drawing overall conclusions, followed by randomized trials and large observational studies that accounted for relevant confounding factors. Adjusted findings were favored over unadjusted associations when both were available. When studies reported conflicting results, differences in study design, patient population, surgical procedure, analgesic technique, and outcome definition were considered, and the inconsistency was reflected in the synthesis. Human perioperative studies were used as the primary basis for clinical conclusions. Experimental pain studies and mechanistic or preclinical evidence were used only to provide biological context and were not considered direct evidence of clinical outcomes.

Because these outcomes are related but not interchangeable, the evidence was considered according to the specific outcome assessed, including postoperative pain intensity, opioid consumption, analgesic efficacy, opioid-related adverse effects, chronic postsurgical pain, persistent postoperative opioid use, and functional recovery. Opioid consumption was not treated as a surrogate for pain severity or opioid sensitivity.

## 3. Biological Sex and Postoperative Analgesic Outcomes

Biological sex may contribute to variability in postoperative pain, opioid consumption, and opioid-related adverse effects. The observed differences are influenced by factors such as age, body composition, preoperative pain, surgical procedure, and analgesic technique.

### 3.1. Postoperative Pain Intensity

Several studies have reported higher postoperative pain scores among female patients, although the magnitude and clinical significance of this association vary considerably [21,22]. In a prospective database analysis of surgical patients, Zheng et al. identified sex-associated differences in pain intensity, pain-related interference, and opioid consumption during the first 24 postoperative hours [23]. However, age and preoperative pain accounted for a substantial proportion of the observed differences, illustrating the difficulty of isolating biological sex from other relevant predictors of postoperative pain [23]. Procedure-specific investigations have similarly reported higher pain scores among female patients in some surgical populations, whereas other studies have found minimal or no clinically important differences after adjustment for baseline characteristics and perioperative treatment [24].

Evidence from regional analgesia further demonstrates this inconsistency. In an analysis of 14,988 adults receiving patient-controlled epidural analgesia after major surgery, Schnabel et al. found that pain scores at rest and during movement were nearly equivalent between female and male patients [25]. Although female patients used less epidural medication, the differences in pain scores were not considered clinically meaningful [25]. These findings suggest that sex-associated differences in postoperative pain may depend partly on the analgesic technique, surgical population, preoperative pain burden, and covariates included in the analysis [26,27]. Biological sex therefore appears to contribute to postoperative pain variability but it is not an independent or consistently reliable predictor of pain intensity.

### 3.2. Opioid Consumption and Analgesic Response

Evidence regarding sex-associated differences in opioid requirements remains mixed. A systematic review and meta-analysis by Pisanu et al., which included 40 comparisons and 6794 patients receiving opioids for acute or chronic pain, found no significant difference between female and male patients in pain reduction 30 min after opioid administration [28]. Female patients, however, self-administered lower daily opioid doses through patient-controlled analgesia [28]. These findings were modified by age, body weight, psychiatric comorbidity, opioid type, patient population, and route of administration, suggesting that biological sex alone does not adequately predict opioid responsiveness.

Studies of postoperative patient-controlled analgesia have produced similarly variable findings. In the patient-controlled epidural analgesia cohort reported by Schnabel et al., female patients used approximately 1.7% to 10.2% less epidural medication over five postoperative days despite reporting similar pain scores [25]. Reduced analgesic consumption therefore cannot necessarily be interpreted as greater opioid sensitivity because it may instead reflect dose-limiting adverse effects.

Procedure-specific studies have also demonstrated that sex-associated differences in pain do not always correspond to differences in opioid consumption. Studies conducted within enhanced recovery pathways, for example, have identified differences in reported pain intensity without corresponding differences in early postoperative opioid use [29]. These findings reinforce the importance of evaluating pain scores, opioid consumption, functional recovery, and adverse effects concurrently rather than using opioid consumption alone as a surrogate for analgesic efficacy.

### 3.3. Opioid-Related Adverse Effects

The most consistently demonstrated sex-associated difference in perioperative care concerns postoperative nausea and vomiting (PONV) [30]. Female sex is an established patient-level predictor incorporated into commonly used PONV risk-assessment models and contemporary consensus guidance [31,32]. This association has been observed across multiple surgical populations, although its magnitude may vary with age, anesthetic technique, history of PONV or motion sickness, smoking status, surgical procedure, and perioperative opioid exposure [32]. Because opioids independently increase PONV risk, female patients receiving opioid-based postoperative analgesia may have overlapping risk factors warranting multimodal prophylaxis [33].

Evidence concerning other opioid-related adverse effects is less consistent. Some studies have suggested sex-associated differences in sedation, pruritus, motor blockade, or respiratory effects, but these findings have not been reproduced uniformly across opioids and analgesic techniques [34]. Observed differences may also be influenced by dose, body composition, age, comorbidities, and route of administration [35]. Current evidence is therefore insufficient to support broadly different monitoring standards based on sex alone. Monitoring should instead reflect the patient’s combined risk profile, including age, frailty, previous opioid exposure, concomitant sedating medications, and the type and dose of analgesia administered.

## 4. Physiological Mechanisms Underlying Sex-Associated Differences in Opioid Response

A growing body of preclinical and clinical research indicates that biological sex influences opioid-receptor function, sensitivity, and downstream signaling, potentially contributing to observed differences in analgesic efficacy and adverse-effect profiles between female and male patients [36,37]. These differences are not simply a function of administered dose but may reflect variation in receptor expression, receptor activation, endogenous opioid signaling, and modulation by gonadal hormones and genetic factors. The following sections describe the principal physiological mechanisms proposed to underlie sex-associated differences in opioid analgesia.

### 4.1. μ-Opioid Receptor Signaling and Neuroanatomy

Experimental neuroimaging studies suggest that painful stimuli may activate the endogenous μ-opioid system differently in female and male participants. Positron emission tomography studies have reported sex-associated patterns of receptor activation or availability in the thalamus, amygdala, basal ganglia, and nucleus accumbens, regions involved in pain processing, stress, and reward [38,39]. Some studies have found greater activation of endogenous μ-opioid neurotransmission among male participants during sustained experimental pain, although the direction and magnitude of these differences vary across brain regions and study populations [38].

These observations should be interpreted cautiously. Receptor availability on positron emission tomography is influenced by receptor density, affinity, and occupancy by endogenous opioids [39]. It is not a direct measure of analgesic sensitivity or the dose of opioid required after surgery. Experimental pain also differs from postoperative pain, which is affected by tissue injury, inflammation, surgical technique, previous pain, and perioperative treatment [40]. Neuroimaging findings provide biological plausibility for sex-associated variation in pain processing, but their ability to predict clinical opioid response has not been established [41].

### 4.2. Hormonal Modulation of Opioid Analgesia

Gonadal hormones may modify pain perception and opioid signaling through their effects on nociceptive processing, endogenous opioid release, and descending pain-modulatory pathways [42]. Estradiol has been associated with changes in μ-opioid receptor activity and pain sensitivity, although its effects appear to depend on hormone concentration, reproductive stage, anatomical region, and the type of painful stimulus studied [43]. Progesterone and testosterone may also influence pain processing, but the available findings are inconsistent [44].

Hormonal variation may contribute to differences observed across the menstrual cycle, pregnancy, menopause, and hormone therapy [45,46]. However, many clinical studies classify participants only as female or male without measuring circulating hormone concentrations or accounting for reproductive status [46]. This limits the ability to determine whether an observed difference is attributable to biological sex, hormonal state, age, or another patient characteristic.

The mechanistic literature supports a possible role for gonadal hormones in opioid-responsive pathways, but it has not established a consistent relationship with postoperative opioid requirements. Hormonal mechanisms should be viewed as one potential contributor to interpatient variability rather than a basis for routine sex-specific analgesic selection.

## 5. Pharmacokinetic Differences Between Female and Male Patients

Sex-associated differences in body composition, hepatic blood flow, gastrointestinal physiology, and renal function may contribute to variability in opioid pharmacokinetics. Their clinical effects are generally modest and overlap considerably between female and male patients. Age, body size, organ function, interacting medications, and previous opioid exposure are usually more informative than sex alone when selecting and titrating perioperative analgesics.

### 5.1. Absorption and Metabolism

Differences in gastric emptying and hepatic enzyme activity have been reported between female and male adults, but their effect on perioperative opioid response is difficult to isolate [47]. Opioid metabolism is also influenced by hepatic and renal function, age, genetic variation, route of administration, and concurrent medications [48]. Because these factors vary substantially among individuals, average sex-associated differences do not reliably predict drug exposure in a particular patient [47,48].

Renal and hepatic function remain more clinically relevant when selecting an opioid [49]. Reduced clearance can prolong opioid effects or permit the accumulation of active metabolites, increasing the risk of sedation and respiratory depression. Medication selection should therefore be based on the patient’s measured organ function and clinical response rather than assumptions derived from sex.

### 5.2. Distribution and Body Composition

Average differences in body composition may affect the distribution of some opioids. Female adults generally have a higher proportion of adipose tissue and lower total body water than male adults of similar body size, although there is substantial overlap between groups [50]. Lipophilic opioids may have a larger apparent volume of distribution in patients with greater adiposity, whereas changes in lean body mass and total body water may influence the distribution of more hydrophilic medications [51]. These relationships are further modified by obesity, aging, frailty, and acute illness [52]. Biological sex is therefore an imprecise substitute for the body-composition and physiologic characteristics that directly influence drug distribution.

### 5.3. Clinical Implications

Available pharmacokinetic evidence does not justify routine sex-specific opioid selection or dosing. Opioids should be titrated according to pain relief, functional improvement, sedation, respiratory status, and adverse effects. Greater attention should be given to renal and hepatic function, age, frailty, body composition, concurrent sedating medications, and previous opioid exposure. Sex remains relevant when considering certain postoperative outcomes, particularly nausea and vomiting, but it should be incorporated into the patient’s overall risk profile rather than treated as an independent dosing variable. This distinction is important: sex-associated pharmacological differences may contribute to variability across populations, but they are not sufficiently consistent to determine treatment for an individual patient.

## 6. Age, Frailty, and Physiologic Reserve

**Figure 1:**
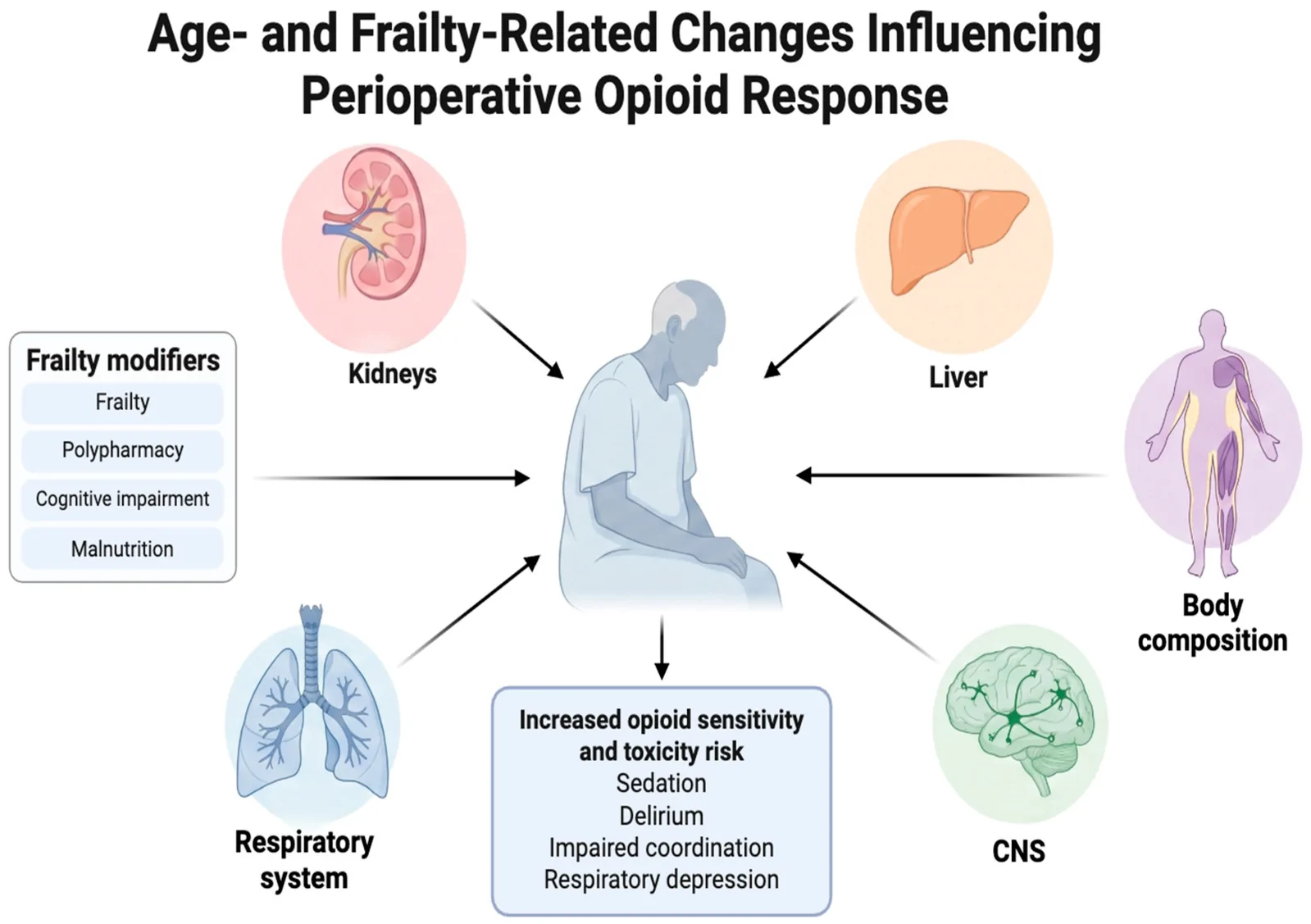
Age- and Frailty-Related Changes Influencing Perioperative Opioid Response.

Age influences postoperative analgesia through changes in pain processing, drug distribution, metabolism, clearance, cognitive function, and susceptibility to treatment-related adverse effects [53]. Older adults frequently present with multimorbidity, polypharmacy, frailty, organ dysfunction, and variable baseline cognition, all of which may complicate postoperative pain assessment and analgesic selection. However, chronological age alone does not adequately describe this clinical heterogeneity. As illustrated in Figure 1, functional status, frailty, cognitive reserve, renal and hepatic function, and previous exposure to analgesic medications may be more informative when developing an individualized perioperative analgesic plan.

### 6.1. Age-Related Differences in Pain Assessment and Postoperative Analgesic Requirements

The relationship between aging and pain perception is complex. Experimental studies have identified age-associated changes in nociceptive thresholds, endogenous pain inhibition, and central pain processing, but these findings vary according to the painful stimulus and study population [54,55]. Older adults may demonstrate reduced sensitivity to some forms of brief experimental pain while simultaneously experiencing impaired endogenous pain modulation, slower resolution of pain, or greater vulnerability to persistent pain [54]. These observations illustrate why chronological age should not be interpreted as a reliable measure of postoperative pain intensity.

Clinical pain assessment may be particularly challenging in older patients with cognitive impairment, delirium, communication difficulties, or sensory deficits [56]. Standard numerical rating scales remain appropriate for many older adults, but patients with impaired cognition may require simplified verbal scales, behavioral pain-assessment instruments, or evaluation of changes in mobility, facial expression, sleep, and participation in care [57]. Failure to recognize pain in cognitively impaired patients may result in undertreatment, whereas nonspecific agitation or behavioral changes may also lead to unnecessary opioid administration if they are incorrectly attributed to pain [57].

Some clinical studies suggest that older surgical patients consume lower quantities of postoperative opioids than younger patients [58]. Lower consumption, however, does not necessarily indicate lower pain intensity or superior analgesia [58]. It may reflect increased opioid sensitivity, reduced patient-controlled analgesia use, clinician concern about adverse effects, altered communication, or greater exposure to dose-limiting sedation and nausea. Accordingly, opioid consumption should not be used in isolation to compare postoperative pain across age groups.

Older adults remain vulnerable to the consequences of inadequately treated pain [59]. Poor pain control can impair coughing, deep breathing, sleep, mobilization, and participation in physical therapy [60]. It may also contribute to sympathetic activation, pulmonary complications, functional decline, and postoperative delirium [61]. Perioperative clinicians must therefore balance the risks of opioid exposure against the harms of undertreated pain rather than assuming that avoidance of opioids is inherently safer.

### 6.2. Age-Related Pharmacokinetic Changes

Aging produces several pharmacokinetic changes directly relevant to perioperative analgesia [62]. Reductions in renal blood flow, renal mass, and glomerular filtration may decrease the clearance of renally eliminated analgesics and active opioid metabolites [62]. Serum creatinine can underestimate renal impairment in older adults with reduced muscle mass [63]. Analgesic selection should therefore be guided by an estimate of renal function and the patient’s overall clinical condition rather than serum creatinine alone [63].

Renal impairment is particularly important for morphine because its glucuronide metabolites are eliminated primarily by the kidneys [64]. Morphine-6-glucuronide retains analgesic activity and may contribute to prolonged opioid effects, whereas accumulation of morphine metabolites can increase the risk of sedation, confusion, and respiratory depression [64,65]. Hydromorphone also produces a renally eliminated metabolite that may accumulate in substantial renal impairment [65]. Opioids without clinically important active renally eliminated metabolites may be preferable in selected patients, but all opioids still require careful titration and monitoring.

Age-related hepatic changes may further affect opioid disposition [66]. Older adults may have reduced liver mass, hepatic blood flow, and metabolic capacity, potentially decreasing the clearance of some hepatically metabolized medications [66]. The clinical effect depends on the hepatic extraction ratio, metabolic pathway, route of administration, and presence of interacting medications [67]. Opioids with high hepatic extraction may be particularly affected by reductions in hepatic blood flow, whereas drugs dependent on specific metabolic enzymes may be influenced by age-related changes in enzyme activity and polypharmacy [66,67].

Body composition also changes with age [68]. Older adults generally exhibit increased adipose tissue, reduced lean body mass, and decreased total body water [68]. Lipophilic medications may therefore have a larger apparent volume of distribution and a prolonged terminal elimination phase, particularly after repeated administration [69]. Conversely, medications distributed primarily in body water may reach higher initial plasma concentrations [68]. Reduced serum albumin in patients with malnutrition or chronic illness can further alter the free fraction of highly protein-bound drugs, although the clinical importance varies by medication [70,71].

These pharmacokinetic changes are not uniform across older adults [72]. Frailty, malnutrition, obesity, renal impairment, hepatic disease, and acute illness may produce greater variability than chronological age itself. Standard doses may therefore result in inadequate analgesia in one older patient and prolonged sedation or respiratory depression in another.

### 6.3. Pharmacodynamic Vulnerability and Opioid-Related Adverse Effects

Age-related pharmacodynamic changes may increase sensitivity to opioids, sedatives, and anesthetic agents even when plasma concentrations are comparable with those observed in younger adults [70]. Reduced physiologic reserve and greater central nervous system sensitivity may allow lower opioid concentrations to produce clinically important sedation, impaired coordination, or respiratory depression [73]. These effects may be amplified by pulmonary disease, renal impairment, and concurrent administration of benzodiazepines, gabapentinoids, antihistamines, muscle relaxants, or other centrally acting medications [73].

Older adults are also more susceptible to constipation, urinary retention, nausea, pruritus, impaired mobility, and falls [74,75]. These adverse effects may delay recovery even in the absence of overt respiratory depression [76]. Medication selection should therefore account for the entire adverse-effect profile rather than focusing exclusively on analgesic potency [76].

Postoperative delirium is an especially important consideration [77]. Baseline cognitive impairment, frailty, sensory impairment, sleep disruption, infection, metabolic abnormalities, pain, and exposure to centrally acting medications may all contribute to delirium [78]. Although opioids can precipitate sedation or cognitive changes, undertreated pain has also been associated with delirium [78]. The relationship is therefore bidirectional and does not support complete opioid avoidance [79]. In a cohort of older adults using postoperative patient-controlled analgesia, patients with delirium continued to experience greater pain despite using similar or greater quantities of opioid medication than patients without delirium [80]. These findings demonstrate that both pain severity and analgesic exposure should be considered when evaluating postoperative cognitive changes.

Certain opioids may pose particular concerns. Meperidine is generally avoided in older adults because its metabolite, normeperidine, may accumulate and produce neurotoxicity [81]. Codeine and tramadol may have unpredictable efficacy because their activity depends partly on CYP2D6-mediated metabolism [82]. Tramadol may additionally contribute to serotonergic toxicity, seizures, and electrolyte disturbances in susceptible patients [83]. These risks should be considered in the context of renal function, pharmacogenomic variability, and concurrent medications.

### 6.4. Frailty as a Modifier of Postoperative Pain and Opioid Response

Frailty describes decreased physiologic reserve and diminished resilience to acute stressors [84,85]. It is distinct from chronological age and may better identify older patients at risk for postoperative complications, functional decline, and medication-related harm [86]. Common frailty assessments incorporate mobility, strength, fatigue, nutritional status, cognition, comorbidity, or the accumulation of health deficits [87]. Although no single instrument has been adopted universally, preoperative frailty assessment can provide clinically useful information beyond age alone.

Emerging studies suggest that frailty may be associated with postoperative pain and opioid consumption, although the direction of this relationship has not been consistent [88,89]. Some frail patients may require greater analgesic support because of pre-existing pain, disability, reduced mobility, or heightened vulnerability to surgical stress [90]. Others may receive or self-administer lower opioid doses because of cognitive impairment, adverse effects, or clinician concern [90]. Frailty should therefore be interpreted as a marker of increased clinical complexity rather than a direct indicator that opioid requirements will necessarily be higher or lower [88].

The interaction between frailty and opioid exposure is clinically important. Frail patients may be particularly vulnerable to sedation, respiratory depression, delirium, falls, constipation, and delayed mobilization [91]. At the same time, inadequate analgesia may impair breathing, mobility, nutrition, sleep, and rehabilitation, further accelerating functional decline. Analgesic plans should therefore be directed toward maintaining function while minimizing treatment-related harm.

## 7. Pre-Existing Chronic Pain and Opioid Exposure

Pre-existing chronic pain and preoperative opioid use are among the most clinically important predictors of difficult postoperative pain management [92]. Patients with chronic pain may enter the perioperative period with altered pain processing, impaired function, sleep disturbance, psychological distress, and previous exposure to multiple analgesic therapies [92,93]. Patients receiving long-term opioids may additionally demonstrate opioid tolerance, physical dependence, or opioid-induced hyperalgesia [93]. These conditions can increase postoperative analgesic requirements while simultaneously increasing susceptibility to sedation, respiratory depression, withdrawal, and persistent postoperative opioid use. These outcomes should be considered separately because greater opioid requirements do not necessarily indicate greater pain intensity, and lower opioid use does not necessarily indicate better pain control.

Chronic pain, opioid tolerance, physical dependence, and opioid use disorder are related but distinct conditions. Tolerance describes a reduced response to a medication following repeated exposure, whereas physical dependence refers to physiological adaptation that can produce withdrawal when the medication is reduced abruptly or discontinued [94]. Neither condition alone establishes opioid use disorder, which is characterized by a maladaptive pattern of opioid use associated with impaired control, continued use despite harm, or other clinically significant consequences [95]. Distinguishing these conditions is essential because stigmatizing or misclassifying a patient receiving appropriately prescribed long-term opioid therapy can undermine communication and result in inadequate perioperative care.

### 7.1. Chronic Pain and Postoperative Outcomes

Pre-existing pain is consistently associated with greater acute postoperative pain and may contribute to the development of chronic postsurgical pain [96]. The effect is not limited to pain arising from the operative site [96]. Patients with chronic pain involving other anatomical regions may also experience greater postoperative pain, suggesting the involvement of generalized alterations in nociceptive processing rather than local tissue pathology alone [97]. Central sensitization, impaired endogenous pain inhibition, sleep disruption, reduced physical function, and psychological distress may all contribute to this vulnerability [98].

Chronic pain may also complicate postoperative pain assessment [99]. A patient’s baseline pain does not disappear following surgery, and postoperative ratings may reflect a combination of pre-existing pain, new surgical pain, anxiety, positioning-related discomfort, and exacerbation of the underlying pain disorder [99]. Assessing only the numerical pain score without considering baseline severity and function may lead either to undertreatment or to escalating opioid administration without meaningful improvement.

Preoperative assessment should therefore establish the location, intensity, and functional consequences of baseline pain; the patient’s usual level of activity; previous responses to analgesic treatments; and realistic postoperative goals. Functional outcomes such as deep breathing, coughing, sleep, mobilization, and participation in rehabilitation may provide more clinically meaningful targets than complete elimination of pain.

### 7.2. Preoperative Opioid Exposure and Tolerance

Patients receiving opioids before surgery frequently require greater postoperative opioid doses than opioid-naïve patients [100]. Repeated opioid exposure produces pharmacological tolerance through adaptations in receptor signaling and downstream neural pathways [101]. As a result, the patient’s chronic opioid regimen may prevent withdrawal and partially treat baseline pain without providing sufficient analgesia for the additional nociceptive burden of surgery [102].

The degree of tolerance varies with the opioid, dose, duration of therapy, adherence, and individual pharmacology [103]. A detailed medication history should document the opioid formulation, dose, route, frequency, duration, prescribing clinician, and timing of the most recent dose. All prescribed and nonprescribed opioids should be considered, including transdermal preparations, combination products, methadone, and buprenorphine. When possible, the total daily regimen may be expressed in oral morphine milligram equivalents to facilitate communication.

Abrupt interruption of long-term opioid therapy can precipitate withdrawal, which may present with anxiety, agitation, diaphoresis, tachycardia, gastrointestinal distress, diffuse pain, and increased sympathetic activity [104]. In most patients receiving conventional long-acting opioid therapy for chronic pain, the established baseline regimen is continued through the perioperative period when clinically feasible [105]. This baseline medication should not be considered sufficient treatment for surgical pain; additional analgesia must be planned according to the procedure and anticipated pain burden [105].

Patients with substantial opioid tolerance may require greater doses of short-acting opioids for breakthrough pain than opioid-naïve patients [106]. However, higher requirements do not eliminate the possibility of opioid-related adverse effects [106]. Respiratory depression, sedation, ileus, nausea, and impaired mobility remain possible, particularly when opioids are combined with benzodiazepines, gabapentinoids, muscle relaxants, or other sedating medications [107]. Opioid administration should therefore be guided by repeated assessment rather than an assumption that tolerance provides complete protection from toxicity.

### 7.3. Opioid-Induced Hyperalgesia

Opioid-induced hyperalgesia is a paradoxical increase in pain sensitivity associated with opioid exposure [108]. Unlike tolerance, in which progressively greater doses may be required to achieve the same analgesic effect, opioid-induced hyperalgesia may manifest as worsening or more diffuse pain despite opioid dose escalation. Proposed mechanisms include N-methyl-D-aspartate receptor activation, increased excitatory neurotransmission, descending facilitation, neuroinflammation, and alterations in spinal nociceptive processing [109,110].

Clinically distinguishing opioid-induced hyperalgesia from tolerance, uncontrolled surgical pain, anxiety, withdrawal, or progression of the underlying pain condition can be difficult. Both tolerance and hyperalgesia may present with greater-than-expected pain and increasing opioid requirements. Pain that becomes more diffuse or worsens following opioid escalation may raise concern for hyperalgesia, but no routinely used perioperative diagnostic test definitively establishes the condition.

Opioid-induced hyperalgesia has been studied in association with long-term opioid exposure and with high-dose or rapidly administered intraoperative opioids, particularly remifentanil [111]. Evidence concerning its frequency and clinical importance remains heterogeneous. When suspected, management may include reducing unnecessary opioid escalation, incorporating nonopioid analgesics, and considering N-methyl-D-aspartate receptor antagonism with ketamine in appropriately selected patients [111,112,113,114]. These strategies should be individualized because postoperative evidence does not support a single universal treatment protocol.

### 7.4. Methadone, Buprenorphine, and Opioid Use Disorder

Patients receiving methadone or buprenorphine require a coordinated perioperative plan. These medications may be prescribed for chronic pain, opioid use disorder, or both, and the indication should be established without judgment. The patient’s regimen should be verified with the prescribing clinician or treatment program when possible.

Contemporary consensus recommendations generally advise against routinely discontinuing buprenorphine during the perioperative period because interruption may increase the risk of withdrawal, destabilization of opioid use disorder, or recurrence of nonmedical opioid use [115,116]. Adequate postoperative analgesia can often be achieved while buprenorphine is continued through multimodal analgesia and, when necessary, additional opioids selected and titrated according to clinical response. The exact regimen may depend on the maintenance dose, anticipated surgical pain, formulation, and availability of acute pain or addiction specialists.

Methadone maintenance therapy is likewise generally continued to prevent withdrawal, but the maintenance dose does not provide complete analgesia for surgery. Additional analgesic treatment may be required. Methadone’s long and variable half-life, potential for accumulation, drug interactions, and association with QT-interval prolongation require careful medication review and monitoring in susceptible patients [117].

Patients with opioid use disorder should receive the same commitment to adequate pain treatment as other patients. Poorly controlled pain, withdrawal, and interruption of established treatment may increase the risk of recurrence or disengagement from care. Whenever possible, perioperative planning should involve the patient, anesthesiology team, surgical team, acute pain service, and the clinician responsible for long-term opioid or opioid use disorder treatment.

## 8. Psychological Factors Affecting Postoperative Pain and Opioid Response

Postoperative pain is influenced not only by surgical tissue injury and analgesic pharmacology but also by psychological factors that shape pain perception, coping, treatment expectations, and recovery. Preoperative anxiety, depression, pain catastrophizing, fear of movement, and broader psychological distress have been associated with greater postoperative pain, impaired quality of recovery, and an increased risk of chronic postsurgical pain. However, these associations vary across surgical populations and do not imply that a patient’s pain is exaggerated or lacks a biological basis. Psychological factors should instead be understood as components of the broader biopsychosocial processes through which pain is experienced and expressed.

### 8.1. Preoperative Anxiety and Depression

Preoperative anxiety is common among patients undergoing surgery and may arise from fear of pain, anesthesia, surgical complications, loss of control, or uncertainty regarding recovery. Higher levels of preoperative anxiety have been associated with greater acute postoperative pain in several surgical populations, although relationships with opioid consumption have been less consistent [118,119]. Anxiety may heighten attention to painful sensations, increase physiologic arousal, disrupt sleep, and reduce the effectiveness of endogenous pain-modulatory pathways [118,119]. These effects may increase the perceived intensity or unpleasantness of postoperative pain without necessarily producing a proportional increase in opioid requirements.

Depression has likewise been associated with poorer postoperative recovery. Patients with depressive symptoms may experience greater pain-related interference, reduced motivation, impaired sleep, and decreased participation in rehabilitation [120,121]. In a prospective study of adults undergoing spine surgery, higher preoperative depression scores were associated with a lower quality of recovery, although anxiety and depression were not independently associated with greater in-hospital opioid consumption [122]. These findings illustrate that psychological factors may influence recovery through outcomes that are not captured by opioid consumption alone.

The relationships among anxiety, depression, pain, and opioid use are likely bidirectional. Chronic pain can contribute to psychological distress, while anxiety and depression may increase vulnerability to severe or persistent pain. Psychiatric medications and associated comorbidities can also influence perioperative management. For example, concurrent benzodiazepine use may increase sedation and respiratory risk when combined with opioids, whereas interruption of established antidepressant or anxiolytic therapy may produce withdrawal or destabilization [123,124]. Medication management should therefore be individualized rather than determined solely by a psychiatric diagnosis.

### 8.2. Pain Catastrophizing

Pain catastrophizing describes a pattern of heightened negative cognitive and emotional responses to anticipated or experienced pain. It is commonly conceptualized through three related components: rumination, magnification, and helplessness. Rumination reflects difficulty disengaging attention from pain; magnification involves anticipating severe or threatening consequences; and helplessness refers to a perceived inability to control or manage the experience.

Higher preoperative catastrophizing scores have been associated with greater acute postoperative pain and an increased risk of chronic postsurgical pain across several surgical populations [125]. A systematic review and meta-analysis examining preoperative anxiety and catastrophizing found that these factors were associated with chronic postsurgical pain, although effect estimates and assessment instruments varied substantially among studies [126,127,128]. Pain catastrophizing may have greater predictive utility than general anxiety in some populations, particularly among patients undergoing musculoskeletal surgery.

Associations with postoperative opioid consumption are less consistent. In a prospective study of adults undergoing spine surgery, greater catastrophizing was associated with higher maximum postoperative pain scores but not significantly greater opioid consumption [122]. Other studies have reported associations between catastrophizing and prolonged postoperative opioid use, particularly when greater preoperative pain is also present [129,130,131,132]. These differing findings again demonstrate that opioid consumption is not a direct measure of pain sensitivity. Medication use may also be influenced by side effects, prescribing practices, patient preferences, and access to nonopioid treatments.

Catastrophizing should not be used as a pejorative label or as a reason to discount a patient’s report of pain. Pain is a subjective experience shaped by interacting sensory, cognitive, emotional, and social processes. A high catastrophizing score identifies a potentially modifiable risk factor and an opportunity for additional perioperative support; it does not demonstrate intentional exaggeration, drug-seeking behavior, or a lack of tissue-based nociception.

### 8.3. Expectations, Fear, and Pain-Related Behavior

Patient expectations may influence postoperative pain and recovery [133]. Expectations regarding the severity of pain, effectiveness of treatment, anticipated disability, and duration of recovery may shape attention, coping behavior, and satisfaction with care [134]. Unrealistic expectations of complete pain elimination can contribute to distress or dissatisfaction even when analgesia is clinically adequate [135]. Conversely, patients who expect that severe pain is unavoidable may delay reporting symptoms or decline potentially helpful interventions [135].

Fear of pain or reinjury may also lead to avoidance of coughing, deep breathing, movement, or physical therapy [136,137]. This pattern can impair functional recovery and reinforce the relationship between pain and disability. Fear-avoidance behavior may be particularly relevant among patients with pre-existing musculoskeletal pain or previous negative surgical experiences [137].

Clear preoperative counseling can help establish realistic expectations. Patients should understand that some postoperative discomfort is anticipated, that pain may be assessed both at rest and with activity, and that the objective of treatment is to support breathing, sleep, mobilization, and recovery while limiting adverse effects. This approach avoids promising complete elimination of pain while reassuring patients that their symptoms will be evaluated and treated.

## 9. Clinical Application of an Individualized Perioperative Analgesic Framework

No single patient characteristic can reliably predict postoperative pain or opioid requirements. In clinical practice, biological sex, age, frailty, chronic pain, opioid exposure, and psychological factors often overlap. Their effects may also work in opposite directions. An older patient receiving long-term opioids, for example, may require more analgesic support because of opioid tolerance while simultaneously having renal impairment, frailty, or respiratory disease that increases the risk of opioid-related harm.

**Figure 2:**
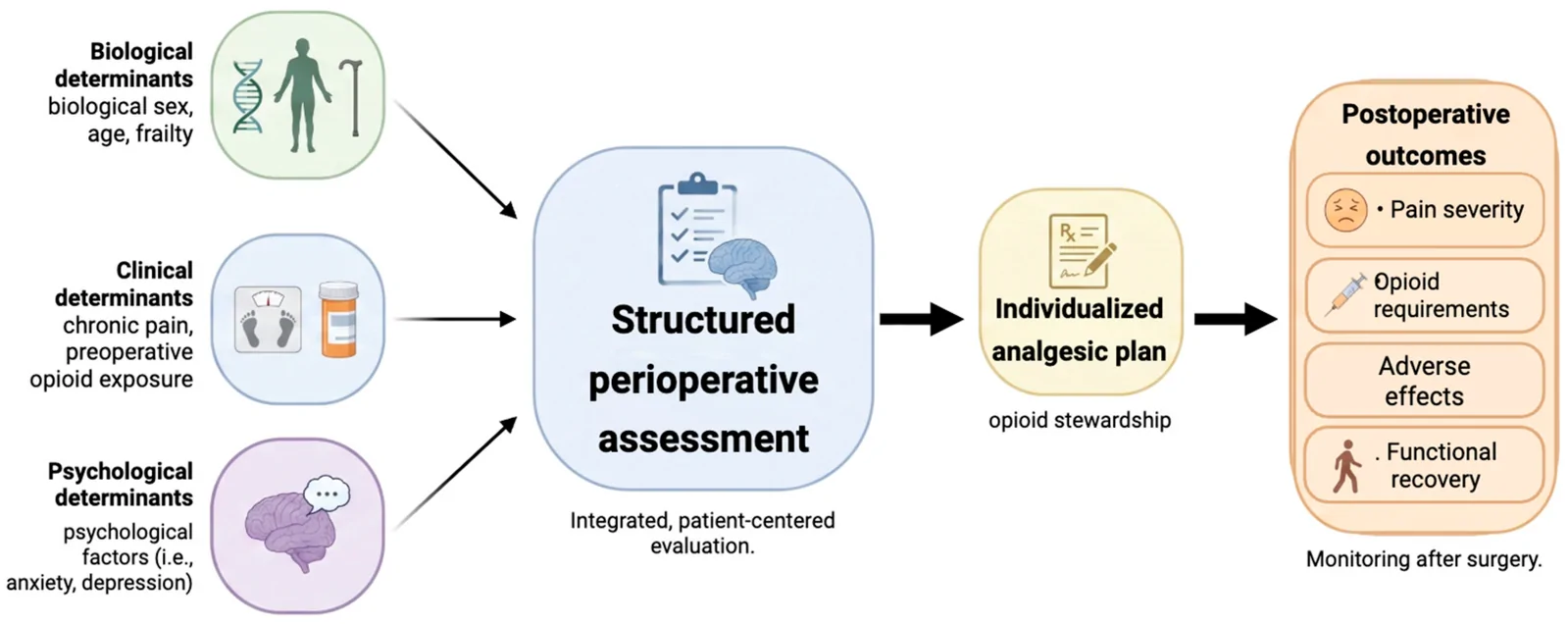
Proposed conceptual framework for individualized perioperative analgesia.

The clinical challenge is therefore not simply identifying which patients will need more opioids. It is recognizing both the expected difficulty of pain control and the patient’s vulnerability to adverse effects. Figure 2 presents a structured approach for incorporating these considerations into preoperative assessment, analgesic planning, and postoperative monitoring. The main factors and their clinical implications are summarized in Table 1.

### 9.1. Preoperative Assessment and Risk Stratification

Preoperative assessment should begin with the patient’s baseline pain and functional status. Clinicians should document the location and intensity of any chronic pain, its effect on mobility and daily activities, and the patient’s previous responses to analgesic treatments. A history of severe postoperative pain, poor response to specific medications, or dose-limiting adverse effects may help guide the perioperative plan. The discussion should also establish realistic goals for recovery, including adequate pain control for breathing, coughing, sleeping, mobilization, and participation in rehabilitation. These patient characteristics may affect several related but distinct outcomes, including pain intensity, opioid consumption, analgesic efficacy, adverse effects, persistent pain, continued opioid use, and functional recovery. Opioid consumption should not be interpreted by itself as a measure of pain severity or opioid sensitivity.

A detailed medication history is particularly important for patients receiving long-term opioids. The formulation, dose, route, frequency, indication, duration of treatment, and timing of the most recent dose should be documented. Methadone, buprenorphine, transdermal preparations, and combination products may require additional planning. Other centrally acting medications, including benzodiazepines, gabapentinoids, muscle relaxants, and sedating antihistamines, should also be identified because their effects may be amplified when combined with opioids.

Chronological age alone provides an incomplete estimate of treatment-related risk. Frailty, cognitive impairment, renal or hepatic dysfunction, respiratory disease, malnutrition, and limited mobility may be more informative when anticipating sedation, delirium, respiratory depression, falls, or delayed recovery. At the same time, concern about these complications should not result in inadequate pain treatment. Poorly controlled pain can also impair breathing, mobility, sleep, and cognition.

Biological sex may contribute to differences in postoperative pain and adverse effects, but it should not be used as an independent basis for opioid dosing. Its clearest clinical application is in established postoperative nausea and vomiting risk assessment. Opioid selection and titration should otherwise be based on the patient’s overall condition and response to treatment.

Psychological factors should be considered without implying that the patient’s pain is exaggerated or primarily psychological. Anxiety, depression, catastrophizing, fear of movement, and negative expectations may identify patients who need additional counseling or perioperative support. Preoperative conversations can help patients understand what level of discomfort to expect and how pain treatment will be balanced with the goals of mobility, safety, and recovery.

Taken together, these findings can be used to estimate two related but distinct concerns: the likelihood of difficult postoperative pain control and the likelihood of treatment-related harm. Some patients will have predominantly increased analgesic needs, whereas others will be more vulnerable to adverse effects. Patients with both high analgesic requirements and high clinical vulnerability may benefit from early involvement of an acute pain service and more frequent postoperative reassessment.

### 9.2. Individualized Analgesic Planning and Postoperative Monitoring

The analgesic plan should remain appropriate for the procedure while accounting for the patient’s risk profile. Regional or neuraxial anesthesia and multimodal analgesia may reduce systemic opioid exposure when clinically appropriate [4,8,9]. However, each component still requires individual consideration. Nonsteroidal anti-inflammatory drugs may be unsuitable in patients with renal dysfunction, cardiovascular disease, or increased bleeding risk. Gabapentinoids may worsen sedation, particularly in older or frail patients and those receiving other centrally acting medications [107]. Ketamine may be helpful in selected opioid-tolerant patients, although its use depends on the anticipated pain burden, relevant contraindications, and available monitoring [111,114].

For most patients receiving conventional long-term opioid therapy, the baseline regimen is continued when clinically feasible to prevent withdrawal and maintain treatment of pre-existing pain [105]. That regimen should not be expected to provide adequate analgesia for the additional pain caused by surgery. Supplemental treatment should be anticipated and adjusted according to the patient’s response. Although opioid-tolerant patients may require larger doses, tolerance does not provide complete protection against sedation, respiratory depression, nausea, ileus, or impaired mobility.

Suzetrigine is a recently introduced nonopioid option for moderate-to-severe acute pain. It selectively inhibits NaV1.8 channels expressed in peripheral nociceptive neurons and acts by preventing the propagation of pain signals up the transmission pathway, a mechanism distinct from opioid-receptor activation [138]. Its peripheral mechanism may make it a useful addition to multimodal analgesia when reducing opioid exposure is an important goal [139]. However, the available trials did not specifically examine its effectiveness in frail, chronically opioid-exposed, or opioid-tolerant surgical patients. Clinically relevant CYP3A-mediated interactions must also be considered [140]. Suzetrigine should therefore be viewed as another potential component of multimodal analgesia, contrasting central acting analgesics like acetaminophen, rather than a substitute for individualized assessment and monitoring.

Postoperative assessment should include more than a numerical pain score. Pain at rest and with movement, sedation, respiratory status, cognition, nausea, mobility, sleep, and participation in rehabilitation provide a more complete picture of treatment effectiveness. A patient who continues to report pain but can breathe deeply and ambulate may require a different adjustment than a patient with the same pain score who cannot cough, sleep, or participate in physical therapy.

Lower opioid consumption does not necessarily indicate adequate analgesia. Medication use may be limited by nausea, sedation, confusion, communication difficulties, or reluctance to request treatment. Conversely, severe or escalating pain should not automatically lead to repeated opioid dose increases. Surgical complications, opioid withdrawal, anxiety, positioning-related discomfort, and worsening of baseline pain should also be considered.

Monitoring should be adjusted as the patient’s condition changes. Older or frail patients may need more frequent assessment of sedation, cognition, respiratory status, and mobility. Opioid-tolerant patients may require greater supplemental doses but remain at risk for adverse effects, especially when opioids are combined with other sedating medications. Treatment should be reassessed after meaningful changes in the analgesic regimen and guided by both pain relief and functional improvement.

### 9.3. Discharge Planning and Opioid Stewardship

Discharge planning should distinguish the patient’s usual medications from those prescribed temporarily for surgical pain. Instructions should explain how nonopioid medications and opioids are to be used, what degree of discomfort is expected during recovery, and when supplemental opioids should begin to be reduced. Clear expectations may help prevent both abrupt discontinuation and unnecessary continuation.

Coordination with the established prescriber is especially important for patients receiving long-term opioids, methadone, or buprenorphine. Communication can reduce conflicting instructions, duplication of prescriptions, and unplanned changes to chronic treatment. Patients with complex chronic pain, substantial opioid tolerance, or opioid use disorder may also require follow-up with the acute pain service, chronic pain clinician, or addiction-treatment team.

The framework presented here is intended as a clinical guide rather than a validated prediction tool. It does not assign a specific analgesic regimen based on sex, age, frailty, opioid exposure, or psychological status. Instead, it encourages clinicians to consider which factors may increase analgesic requirements, which may increase the risk of adverse effects, and when both concerns are present. This approach may provide a more practical basis for perioperative planning than relying on any single patient characteristic.

## 10. Conclusions

Postoperative pain and opioid responsiveness are influenced by a complex interaction of biological, clinical, and psychological factors rather than any single patient characteristic. Biological sex, age, frailty, chronic pain, preoperative opioid exposure, and psychological status each contribute to variability in analgesic requirements and opioid-related adverse effects, but their combined effects are more clinically relevant than any individual factor. An individualized approach to perioperative pain management should therefore combine these patient-specific factors with procedure-specific considerations to optimize analgesia while minimizing treatment-related harm. Current evidence supports comprehensive preoperative assessment, multimodal analgesia, and ongoing clinical reassessment as the foundation of individualized perioperative pain management. However, the available evidence remains heterogeneous across patient populations, surgical procedures, analgesic techniques, study designs, and outcome definitions. The integrated framework presented in this review is conceptual and requires prospective validation before it can be used as a clinical prediction tool. Future research should focus on developing integrated risk prediction models that combine multiple patient-specific determinants to better guide analgesic selection. Such approaches have the potential to improve pain control, enhance recovery, reduce opioid-related adverse effects, and support more patient-centered perioperative care.

## Figures and Tables

**Table 1:** Integrated patient-specific determinants of postoperative pain and opioid response: Implications for individualized perioperative analgesia.

Patient-Specific Factor	Effect on Postoperative Pain	Effect on Opioid Response	Clinical Considerations
**Biological Sex**	Variable; females often report higher pain scores, although findings are inconsistent	No consistent difference in opioid efficacy; female sex associated with greater PONV risk	Do not alter opioid dosing based on sex alone
**Age**	Pain perception and reporting may differ with aging	Increased pharmacodynamic sensitivity; lower opioid doses may produce greater adverse effects	Individualize dosing according to physiological reserve rather than chronological age
**Frailty**	May increase vulnerability to poor recovery and functional decline	Increased risk of sedation, delirium and falls	Assess frailty preoperatively and titrate analgesia cautiously
**Chronic Pain**	Associated with greater acute postoperative pain and increased risk of chronic pain	Higher analgesic requirements; baseline pain complicates assessment	Document baseline pain and functional status; emphasize multimodal analgesia
**Preoperative Opioid Exposure**	Greater postoperative pain and opioid requirements	Tolerance, dependence, possible opioid-induced hyperalgesia	Continue baseline therapy when appropriate and anticipate additional analgesic requirements
**Psychological Factors**	Anxiety, depression, and catastrophizing may increase pain intensity	Variable relationship with opioid consumption	Provide counseling, expectation management, and individualized perioperative support

## Data Availability

No new data were created or analyzed in this study. Data sharing is not applicable to this article.
